# Achieving Research Impact Through Co‐creation in Community‐Based Health Services: Literature Review and Case Study

**DOI:** 10.1111/1468-0009.12197

**Published:** 2016-06-06

**Authors:** TRISHA GREENHALGH, CLAIRE JACKSON, SARA SHAW, TINA JANAMIAN

**Affiliations:** ^1^ Nuffield Department of Primary Care Health SciencesUniversity of Oxford; ^2^ Discipline of General Practice, School of MedicineUniversity of Queensland

**Keywords:** co‐creation, knowledge production, health research systems

## Abstract

**Policy Points**:

- Co‐creation—collaborative knowledge generation by academics working alongside other stakeholders—is an increasingly popular approach to aligning research and service development.
- It has potential for “moving beyond the ivory towers” to deliver significant societal impact via dynamic, locally adaptive community‐academic partnerships.
- Principles of successful co‐creation include a systems perspective, a creative approach to research focused on improving human experience, and careful attention to governance and process.
- If these principles are not followed, co‐creation efforts may fail.

**Context:**

Co‐creation—collaborative knowledge generation by academics working alongside other stakeholders—reflects a “Mode 2” relationship (knowledge production rather than knowledge translation) between universities and society. Co‐creation is widely believed to increase research impact.

**Methods:**

We undertook a narrative review of different models of co‐creation relevant to community‐based health services. We contrasted their diverse disciplinary roots and highlighted their common philosophical assumptions, principles of success, and explanations for failures. We applied these to an empirical case study of a community‐based research‐service partnership led by the Centre of Research Excellence in Quality and Safety in Integrated Primary‐Secondary Care at the University of Queensland, Australia.

**Findings:**

Co‐creation emerged independently in several fields, including business studies (“value co‐creation”), design science (“experience‐based co‐design”), computer science (“technology co‐design”), and community development (“participatory research”). These diverse models share some common features, which were also evident in the case study. Key success principles included (1) a systems perspective (assuming emergence, local adaptation, and nonlinearity); (2) the framing of research as a creative enterprise with human experience at its core; and (3) an emphasis on process (the framing of the program, the nature of relationships, and governance and facilitation arrangements, especially the style of leadership and how conflict is managed). In both the literature review and the case study, co‐creation “failures” could often be tracked back to abandoning (or never adopting) these principles. All co‐creation models made strong claims for significant and sustainable societal impacts as a result of the adaptive and developmental research process; these were illustrated in the case study.

**Conclusions:**

Co‐creation models have high potential for societal impact but depend critically on key success principles. To capture the nonlinear chains of causation in the co‐creation pathway, impact metrics must reflect the dynamic nature and complex interdependencies of health research systems and address processes as well as outcomes.

This article addresses co‐creation, which we define as the collaborative generation of knowledge by academics working alongside stakeholders from other sectors. Our particular interest, and the focus of our case study, is community‐based research collaborations, though we refer in passing to other forms of co‐creation. We consider the extent to which co‐creation models linking university academics with health services in their local community might help solve the well‐described issue of “ivory tower” research that (for whatever reason) is not implemented, leading to waste^1^ and an entrenchment of the “two cultures” problem—that is, of researchers and research users failing to understand or engage with one another.^2^ To put that question another way, we address the hypothesis that because of its emphasis on civic engagement, intersectoral collaboration, power sharing, and ongoing conflict resolution, co‐created research might have particularly strong and enduring impact on health and wider outcomes in the local or regional setting in which universities are located.

The impetus for this article was a wider systematic review of the literature, funded by the UK Health Technology Assessment Programme, which asked, What conceptual or methodological approaches to assessing the impact of programs of health research have been developed and/or applied in empirical studies? That review, whose methodology, search strategy, and findings are described in detail elsewhere,^3^ included a systematic search of 8 electronic databases (including grey literature) plus hand searching and reference checking. It identified more than 20 different models and frameworks for research impact and 110 studies describing their empirical applications. A number of these models (described variously as “realist,” “participatory,” or “co‐production”) shared an element of collaborative knowledge generation and focused on local or regional university‐community partnerships. They were described only briefly in the original systematic review (which focused mainly on conventional clinical trials and impact through knowledge translation). In addition, the lead author reviewed collaborative models of impact in more detail in an unpublished dissertation.^4^

This article presents the findings of the above work relating to co‐creation between university academics and their local communities and applies them to an illustrative empirical case study. It is structured as follows. First, we summarize and critique what is known as the “Mode 2 hypothesis,” which depicts a relatively recent shift in the relationship between universities and society from knowledge translation (or utilization) to knowledge production (or co‐creation). Second, we review 4 contrasting models of co‐creation that have relevance to university‐community partnerships—“value co‐creation” in the business and management literature, “experience‐based co‐design” in design science, “technology co‐design” in computer science, and “participatory research” in community development. We suggest that, despite their different origins and ideological allegiances, all share important philosophical assumptions and operating principles. Third, we review a somewhat sparse literature on the interorganizational structures developed to support co‐creation between university academics and their local community partners. Fourth, we consider the question of research impact and the mechanisms by which this may be achieved in co‐creation models. Fifth, we present an empirical example of co‐creation in practice from a major primary care development project in Queensland, Australia. We conclude by underlining the key elements that appear essential to maximizing impact in university‐community co‐creation partnerships.

## Mode 2: From “Knowledge Translation” to “Knowledge Production”

In most medical fields, the dominant assumption about knowledge is that it exists (or could exist) as more or less generalizable facts about the world. Research impact is viewed as occurring via translation (or utilization) of these facts, depicted at 4 levels: individual (eg, via change in practitioners’ knowledge or attitudes), interpersonal (eg, via peer influence), collective (eg, via prevailing professional opinion and ethical codes), and organizational (eg, via roles, routines, or institutional constraints).^5^

Knowledge translation research (“implementation science”) is a diverse intellectual community but tends to focus on a search for transferable facts about what works at each of these 4 levels.^6^ It has, for example, revealed what academics can do to make their research outputs more accessible and usable by clinicians and policymakers. Such strategies include “tailoring,” “targeting,” “framing,” and “narrativizing” one's message; mobilizing “boundary spanners,” “brokers,” and “champions”; providing clear estimates of the strength and quality of evidence and any residual uncertainties; training service staff to find and evaluate research evidence; providing incentives and administrative support for knowledge transfer activities in organizations; and engaging the media.^6^, ^7^, ^8^, ^9^, ^10^, ^11^

All these strategies are important. But they reflect a somewhat determinist, evidence‐into‐practice logic, with its metaphors of producer‐push and demand‐pull.^12^ This logic has been critiqued by social scientists as both empirically unfounded^13^, ^14^, ^15^, ^16^, ^17^ and philosophically and ideologically flawed.^18^ In short, the concept of knowledge translation is predicated on the assumption that research knowledge is created by university‐based scientists and *then* packaged and processed in a way that makes it accessible to nonacademics. Yet the (partly medical, partly social) science of applied health research rarely conforms to this linear sequence.

In 1994, a book titled *The New Production of Knowledge* introduced a new taxonomy: “Mode 1 scientific discovery” and “Mode 2 knowledge production.”^19^ Mode 1 refers to the conventional model of university‐based research that is then “translated.” Gibbons and colleagues describe this mode as “hegemonic” (that is, relating to domination) and driven by closed hierarchies of scientists and their universities, implicitly at the expense of nonacademic stakeholders. Mode 2 knowledge, in contrast, is “*socially distributed, application‐oriented, trans‐disciplinary and subject to multiple accountabilities*.”^20^ ^(p179)^ Such knowledge is generated within its context of application—a heterogeneous transaction space embracing university, state, economy, culture, and the wider public sphere. In this space, problems are identified, questions debated, methodologies developed, and outcomes disseminated. There are many players, many experts (of different kinds), and an evolving collective view (though rarely a consensus) on what the questions and challenges are. To be credible with its diverse audiences, Mode 2 must be seen as socially as well as scientifically robust (hence ethical, environmentally sustainable, socially inclusive, and an appropriate use of public resources).

In Mode 2, a range of theoretical perspectives and practical approaches—including but not restricted to specialist scientific techniques—are mobilized and managed, often for a limited period only, to address a particular set of problems. Planning, execution, dissemination, and implementation of research are not separate and linear phases but interwoven, and the relationship between scientists and research users (industry, policymakers, citizens, and so on) is one of co‐production rather than producer‐consumer or contractor‐commissioner.

In a second book, three of the original authors explained that Mode 2 emerged in parallel with increasing complexity and uncertainty in both science and society.^21^ In the past 30 years in particular, science has become much less certain: “*its composition more heterogeneous, its values more contested, its methods more diverse and its boundaries more ragged*.”^21^ ^(p2)^ In the 1970s, an emphasis on control and predictability in both politics and science, which was depicted as potentially able to fix the problems of society, gave way to a growing recognition of the nonlinearity and inherent unpredictability of both social and scientific phenomena.^21^

Using the philosophical lens of pragmatism, Van de Ven and Johnson argue that Mode 2 is essentially a dialectical process of bringing competing perspectives (academic and practical) to bear on a problem^22^—a process others have called “bricolage.”^23^ They explain: “*By exploiting multiple perspectives, the robust features of reality become salient and can be distinguished from those features that are merely a function of one particular view or model*.”^22^ ^(p815)^ Such an approach is invariably power‐charged and conflict‐ridden; the key to its success is making power relations explicit and encouraging task‐oriented conflict (which can be creative and productive) while managing the potentially destructive influence of interpersonal conflict.

While the Mode 2 hypothesis is appealing, the original authors offer only sketchy empirical examples, mostly from outside health care. An alternative interpretation of the university‐society link is that instead of a progressive shift from Mode 1 (university‐based, needing “translation”) to Mode 2 (collaboratively generated in its field of application), these modes have coexisted for decades, along with an age‐old tension between scientific rigor and societal relevance.^24^, ^25^ But whilst there is no doubt that university academics have long collaborated with both industry and government on particular projects (reviewers of an earlier draft of this article, for example, mentioned the development of radar, the internet, nuclear weapons, and the breaking of the Enigma code in World War II), there is also strong evidence from the “research on research” literature that, overall, health research in both the United States and the United Kingdom involves increasingly complex intersectoral networks in which university scientists engage with policymakers, civil society, and industry to a far greater extent than in the past.^26^

The Mode 1/Mode 2 taxonomy was developed in Europe; a comparable analytic framework (“technocratic” versus “democratic” models of community engagement by universities) was proposed in the United States by Jameson, Clayton, and Jaeger.^27^

## Models of Co‐creation in Health Care

Whilst the term “Mode 2” is rarely used in the health care literature, it describes a number of research approaches that are gaining ground and that are more commonly referred to as “co‐creation” or “co‐production.” Table 1 summarizes 4 models of co‐creation, which (to our knowledge) emerged largely independently of one another. The list is not exhaustive. For example, since the empirical focus of this article is community‐based research, we have omitted human factors in patient safety research (a largely hospital‐based co‐creation approach),^28^ collaborative approaches to evaluation,^29^ and various approaches to the collaborative design of software.

**Table 1:** Different Models of Co‐creation

				Key Stakeholders
	Parent	Driving		in the Co‐creation
Model	Discipline	Principles	Goal	Process
1. Value co‐creation^30^, ^31^	Business and management	People are naturally creative and seek to generate value for themselves and others. Value is created by providing platforms that allow stakeholders to interact and share their experiences. Value is subjective (ie, it depends on individuals’ experience of what is created) and takes many forms.	Developing long‐term stakeholder partnerships Building “ecosystems of capabilities” across private, public, and social sectors Increasing creativity, productivity, and growth Improving the value of co‐created products and services	Customers, staff, suppliers, government, partner organizations, funders, end users, citizens
2. Experience‐based co‐design^32^	Interdisciplinary (phenomenology, design science, management)	The patient experience is the starting point for redesigning a health service. Patients and staff can work together on the redesign process.	Improved patient experience of health services	Patients, staff, facilitators
				
3. Technology co‐design^33^, ^34^	Computer science	The starting point for technology design is the intended users’ capabilities and what matters to them.	Technologies that are acceptable, fit for purpose, and which support effective and efficient work processes	Technology users and carers, technology designers, support staff
		Technologies are never “plug and play”; helpdesk and service support must be designed in parallel with the technology itself.		
4. Community‐based participatory research^35^, ^36^	Development studies	Power imbalances between researchers and community members must be recognized and addressed. Sustainable change depends on mutual trust, built over time through shared endeavor.	Local learning and change that reduce inequalities Generalizable principles about effective partnerships	Vulnerable communities, advocates, researchers
				

Some of the models in Table 1 are more explicitly research‐oriented than others, though all have been used in community‐based health research. They have significant differences in perspective and ideology.

**Figure 1:**
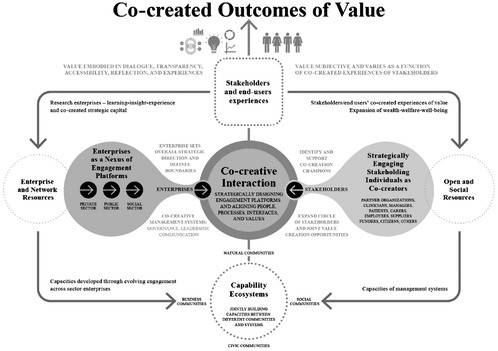
Value Co‐creation^a^ ^a^Adapted from Figures 1, 2, 3 in Ramaswamy and Ozcan.^37^ ^(p29)^

Value co‐creation (Figure 1 and model 1 in Table 1), which originated in the business and management field, is focused on creating value (both economic and otherwise) with and for all stakeholding individuals, with the goal of developing sustainable long‐term partnerships and enhancing economic and societal benefits: “*Co‐creation is joint creation and evolution of value with stakeholding individuals, intensified and enacted through platforms of engagement, virtualised and emergent from ecosystems of capabilities, and actualised and embodied in domains of experiences, expanding wealth‐welfare‐wellbeing*.”^37^ ^(p14)^

Experience‐based co‐design (model 2 in Table 1) was developed by Bate and Robert in the health services research field, who drew on phenomenological philosophy, design science, and management studies with a view to ensuring that health services and/or care pathways were designed and continually redesigned around the experiences of patients and carers. Key features of this model include a grounding in phenomenology (the perceptions and emotional reactions of the individual patient), its strong focus on pragmatic application in frontline health services,^32^ and the use of collective sensemaking and negotiation to “*[produce] new understandings, relationships, and engagements*.”^38^ ^(p73)^ To that end, its original architects, supported in the United Kingdom by medical charities, have developed tools, training programs, and manuals to be used by frontline service managers and facilitators. The approach is described thus in one online manual:

*[Experience‐based co‐design] involves gathering experiences from patients and staff through in‐depth interviewing, observations and group discussions, identifying key “touch points” (emotionally significant points) and assigning positive or negative feelings. A short edited film is created from the patient interviews. This is shown to staff and patients, conveying in an impactful way about how patients experience the service. Staff and patients are then brought together to explore the findings and to work in small groups to identify and implement activities that will improve the service or the care pathway*.^39^

Experience‐based co‐design is gaining popularity in the United Kingdom and has been widely used in hospital‐based quality improvement efforts (see review by Donetto and colleagues^40^). Its use in community‐based improvement projects has been much more limited (Table 2). The approach appears to be extremely effective in capturing narratives and thereby identifying key “touch points,” but as the examples in Table 2 illustrate, some projects have failed to follow through to significant and sustainable redesign as a result of these.^41^

**Table 2:** Empirical Examples of Co‐creation in Community‐Based Health Care

Lead Author		Brief		
(Country)	Goal	Description	Key Outcomes	Comment
**1. Value co‐creation**				
Jackson^45^ (Australia)	To support and extend the capacity of primary health care locally and better integrate service delivery across the sector	Development of a “beacon” primary health care practice with shared governance between university, local health economy, and community	Within 3 years, new practice was revenue neutral; complex care shifted from hospital to community; metrics of process and outcome for chronic disease management improved	See detailed description in text.
**2. Experience‐based co‐design**				
Larkin^46^ (UK)	To use the service user experience to improve mental health services	Combined community and hospital service; user and staff interviews; co‐design event focusing on “touch points”	Priorities for improvement (eg, pathways in and out of hospital) identified and addressed by a series of collaborative redesign working groups	Whilst priorities for redesign were readily identified, many were unimplemented at 9‐ and 18‐month review.
Pearce^47^ (UK)	To use the experience of service users and staff to improve sexual health services	Community‐based sexual health clinics in multiethnic inner London borough; “mystery shopper” sexual health patients and staff workshops	More client‐centered ethos, shorter waiting times, improved physical environment	Challenges included logistics and identifying and retaining a “representative” group of service users.
**3. Technology co‐design**				
Clemensen^48^, ^49^ (Denmark)	To improve the design and delivery of a technology‐supported, community‐based service for diabetic foot	User experience workshops followed by design workshops with testing of prototypes in the home and then further field testing with new users	Prototype of technological and service solution for remote follow‐up of diabetic foot problems	The design process appeared successful and proof of concept was demonstrated in a small sample, but follow‐through to service change was not reported.
Vassilakopoulou^50^ (Norway)	To improve booking of outpatient appointments in Norwegian health care (2 case studies)	Combined technology co‐design and experience‐based co‐design	Workable electronic booking service for health care providers	The co‐design process happened slowly and took much effort: “*[I]t was gradually realized what type of relationship the booking process entailed, and what is needed in order to put in place an electronic service to support this relationship*” (page 202).
Wherton^34^ (UK)	To inform design of telehealth/telecare services and technologies for older people with assisted‐living needs	Preliminary ethnographic phase followed by workshops with users, providers, and industry and then one final combined workshop	Input to service improvement and industry [re]design of telecare technologies; general principles for technology/service co‐design for this user group	Mismatches between current technologies/services and user needs were evident, but significant service change was not achieved within the timescale and resources of the study.
**4. Community‐based participatory research**				
Potvin,^51^ Nield^52^ (Canada)	To prevent type 2 diabetes in a high‐risk indigenous community using participatory approaches	Co‐design with community members targeting food outlets and actions, and utilization of exercise facilities	General principles for ethical and democratic academic‐community partnerships; sustained partnership over 20+ years with evolving program of community‐based resources and facilities	Despite exemplary processes, changes in hard outcomes (eg, significant reduction in diabetes incidence) were difficult to demonstrate, partly due to multiple confounders.
Findley^53^, ^54^ (USA)	To reduce ethnic and socioeconomic differences in child immunization rates through community participation	Participatory approach emphasizing community leadership, integration with existing community programs, parental empowerment, peer health educators, tracking and feedback, and links with health providers	High parental satisfaction with program; increased immunization rates that were significantly higher than national average, especially for minority groups	Success was attributed to community ownership, integration with existing programs, peer educators, intense parental education and empowerment, and reminders.

Technology co‐design (model 3 in Table 1) originated at the cusp of computer science and management studies in the 1950s and was based on sociotechnical systems theory. It proposes that technologies and work practices are best co‐designed using participatory methods in the workplace setting, drawing on such common‐sense guiding principles as staff being able to access and control the resources they need to do their jobs and insisting that processes should be minimally specified (eg, stipulating ends but not means) to support adaptive local solutions.^42^ This early work inspired the emergence of an interdisciplinary field of inquiry known as computer supported cooperative work (CSCW), a central focus of which is the workarounds that people develop, individually and collaboratively, to overcome what has been termed the “brittleness” of software and other technologies.^43^ As with experience‐based co‐design, most examples of technology co‐design in health care are hospital‐based. Emerging community‐based examples illustrate an effective design process but often also reveal practical or logistical difficulties with following through to sustainable service change (see Table 2).

Community‐based participatory research (CBPR, model 4 in Table 1), which originated in the development studies literature, defines its goals in terms of human welfare and emphasizes equity and social justice: “*Community‐based participatory research is an orientation to research that emphasizes ‘equitable’ engagement of partners throughout the research process, from problem definition, through data collection and analysis, to dissemination and use of findings to help effect change*.”^35^ ^(p1615)^A closely related approach described by Dostilio^44^ and cited in the higher education literature is titled “democratically engaged partnerships” and emphasizes power sharing, reciprocity, and mutual learning between a university research group and a local community partner. Unsurprisingly, CBPR has been widely used in community‐based public health programs; 2 examples are listed in Table 2.

Despite their significant differences in perspective and ideology, the 4 co‐creation models have a number of common features. First, they all take a *systems perspective*, depicting (in different ways) multiple interacting entities that are emergent, locally adaptive, self‐organizing, and path‐dependent, and which generate outcomes that cannot be fully predicted in advance.

Second, they view research as a *creative endeavor*, with strong links to design and the human imagination. Design, especially in relation to business processes and technologies, can be thought of as part science, part art—and in both cases, it requires imagination, exploration, field testing, and reflection on emerging data to move from idea to prototype to the refined output (product, process, or service). All the models place *individual experience* (especially that of the patient, but also of staff) at the heart of this creative design effort. Indeed, Bate and Robert have emphasized that it is the *experience*—and particularly the “emotional touch points”—that needs to be designed, not the process (an efficient process may make for a poor patient experience and vice versa).^32^

Third, all the approaches listed in Table 1 recognize (to a greater or lesser extent) that the *process* of co‐creation is as important as any particular products or services generated. This includes how the project or program is set up and framed, including how different partners view the co‐creation process; the nature of relationships (which require respect and reciprocity); and governance and facilitation arrangements, especially how conflict is managed and the style of leadership—“*leaders who advance a democratic orientation and who promote structures and facilitation techniques that create space for transparency, deliberation, and inclusion of diverse stakeholders*.”^44^ ^(p241)^

All the co‐creation models in Table 1 make strong claims that because of their developmental and adaptive approach, outputs are more likely to be fit for purpose, acceptable, valuable, and enduring than the outputs of a comparable effort organized to conventional, “logic model” principles.

Co‐creation research raises important questions about the relationship between (the generation of) knowledge and (the distribution of) power. In a *Nature* editorial, Ziman expressed concern that in multi‐stakeholder partnerships, scientists would be pressured by government, industry, lobbying groups, and so on to shape their research and interpret their findings in particular ways (thereby distorting the scientific process).^25^ Whilst some authors have written positively about the entrepreneurial university^55^ and the “triple helix” of evolving university‐industry‐government knowledge production,^56^ others have warned of the dangers of “academic capitalism,” which include (but are not limited to) overt conflicts of interest,^57^ and proposed adding “Mode 0” (“*knowledge production based on relations of power and patronage*”) to Gibbons and colleagues’ original taxonomy.^58^ The evidence base on this important controversy, which is beyond the scope of this article, has been reviewed by others.^59^

In some community‐based models of co‐creation, especially experience‐based co‐design (with patients and carers) and CBPR (with vulnerable communities), a central issue is the nonacademic partner's *lack* of power. Power remains an issue (but is generally less salient) in technology co‐design, in which the community partner's primary role is that of technology consumer (and, depending on the business model, perhaps customer), and in value co‐creation, in which the main role of the community stakeholder(s) is “partner(s) in the value chain.” In each case, there are inherent power differentials, and the end user will need advocacy support and power‐sharing governance arrangements to participate meaningfully in the co‐creation process. Baranick and colleagues, writing in the global health literature, offer a model for transitioning from CBPR (in which the community partner is depicted as lacking the capacity to assimilate knowledge and handle operational activities on its own) to co‐creation of value (in which the community partner has matured in absorptive capacity and dynamic capability, and is hence in a stronger position to negotiate).^60^

## Structures Supporting Co‐creation

The growing popularity of co‐creation in some circles in recent years should be seen as part of a wider change in the science‐society relationship. In particular, the simple, one‐way, and readily auditable relationship between a group of scientists that undertakes research and a funder that commissions and then uses such research (to the extent that this simple relationship ever existed in the first place) has given way to complex networks of intersectoral collaborations and interdependencies, and to an ongoing debate about what should be researched, by whom, and how.

As Nowotny and colleagues commented: “*The research process can no longer be characterised as an ‘objective’ investigation of the natural (or social) world, or as a cool and reductionist interrogation of arbitrarily defined ‘others.’ Instead it has become a dialogic process, an intense (and perhaps endless) ‘conversation’ between research actors and research subjects*.”^20^ ^(p187)^ Today, we might use the term “research stakeholders” to extend this sentiment to those (such as policymakers, knowledge intermediaries, fundraisers, or citizen activists) engaged in different ways in the research process.

The complex forms in which people and organizations negotiate, undertake, and implement research are sometimes referred to as “health research systems”—defined as organized networks of researchers and other stakeholders who provide a context for health sciences research and its uptake and application^26^, ^61^ or (to the extent that the partners identify as an entity and have a formal structure and governance arrangements) as multi‐stakeholder health research collaborations.^62^

Such collaborations, which often align with one or more co‐creation models listed in Table 1, include:

- Canada's Community‐University Research Alliances, including universities, community organizations, schools, health and social care providers, and citizens^63^;
- The Netherlands’ Academic Collaborative Centres for Public Health, including universities, policymakers, and local public health organizations^64^;
- Australia's Centre of Research Excellence in Quality and Safety in Integrated Primary‐Secondary Care (described in the case study below)^65^; and
- The United Kingdom's Collaborations for Leadership in Applied Health Research and Care (CLAHRCs), comprising universities, local health and social care organizations, and citizens^66^; and Academic Health Science Networks (AHSNs), comprising universities, industry and commercial partners, and health care organizations.^67^

With the exception of AHSNs, whose main focus is on basic science research and its translation to bedside tests and treatments (and hence, on strong industry partnerships and commercialization opportunities), all these multi‐stakeholder research collaborations are primarily community facing and have strong representation from primary health care and social services. All are designed to draw researchers and end users together earlier and more powerfully than in traditional research translation models. Academics, service users, and service organizations work together from the outset to frame locally relevant research questions, create research designs that reflect “real‐world” environments, and commit to both implementing the research and utilizing its findings in the broader health service delivery community. Many (though not all) prioritize the pursuit of social justice (eg, reduction of inequalities in access to services) and/or the development of research capacity in health care organizations and community partners.^36^, ^62^

## Research Impact in Co‐creation Models

In many countries, notably the United Kingdom where the 2014 Research Excellence Framework sought evidence of “impact” in all academic disciplines,^68^ intersectoral outreach along with targets for deliverables beyond academia (such as improved health and well‐being, patents and profits for commercial partners, financial savings for patients or the public purse, greater public understanding of science, cultural artifacts, and so on) is becoming a dominant component of universities’ core business.^69^, ^70^

A key driver for research impact is researchers’ relationships with different stakeholder groups—including industry, policymakers, health care providers, service users, the media, and citizens. It has long been known (though not always acted upon) that proactive linkage and exchange introduces researchers and the intended users of research to one another's worlds, builds two‐way bridges between them, and develops the mutual trust on which collaboration depends.^71^, ^72^ This finding resonates with Weiss's taxonomy of mechanisms by which research evidence influences policy—more often through a steady process of mutual enlightenment born of long‐standing exposure to each other's ideas than through the direct and instrumental use of published research evidence.^73^

“Mode 1” research impact frameworks take a more or less linear view of impact (dollars in, grants awarded, papers published, findings translated, impact achieved) and generally focus on a limited range of predefined impact metrics such as deaths avoided or improved health status.^3^, ^74^ Such “logic models” have their place, but they are particularly unfit for purpose for assessing the interactions, negotiations, and activities of an unstable and organically evolving research system in which the chain of causation for any particular outcome is diffuse and contested.^75^, ^76^ It follows that as the complexity of health research systems increases, there is limited mileage in attempting to measure downstream impacts of co‐created research. Conversely, much could be learned about impact in such complex systems by shifting the focus of study to *the processes by which knowledge is collaboratively generated*.

An emerging science of “societal impact assessment” seeks to categorize and measure both the various interactions between university academics and other stakeholders and the outputs and outcomes emerging from them.^77^, ^78^ The European Seventh Framework Programme, for example, sought to assess synergies with science education, engagement with civil society and policymakers, dissemination to the general public in multiple languages, and the employment consequences of the research.^79^ But measures of societal impact have had limited success in practice, partly because stakeholders rarely agree on what should be measured or how^80^ and partly because (some have argued) this conventional framing of research impact is predicated on a technocratic, “intellectual property” view of knowledge and overlooks more critical perspectives on the relation between knowledge generation and the distribution of power among stakeholders.^44^, ^57^, ^59^, ^81^, ^82^

Boaz and colleagues describe examples of co‐created research from the international development literature, which has traditionally favored “*qualitative, participatory evaluations with a focus on learning and service improvement*.”^83^ ^(p260)^ Naturalistic methods such as ethnography and storytelling have been used to capture multiple voices in what are typically presented as “positive utilization narratives” that describe the processes of engagement as well as key outputs and outcomes. But as pressure to demonstrate accountability grows, sponsors increasingly question the veracity of such narratives and seek external evaluations that privilege logic models, quantitative methods, and predefined performance indicators.

**Figure 2:**
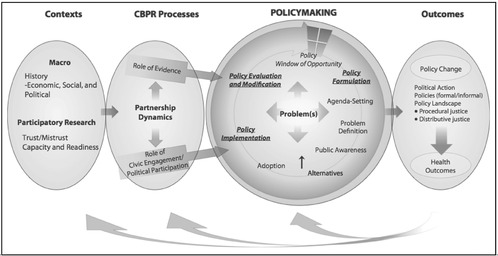
Cacari‐Stone and Colleagues’ Model of Impacts From Community‐Based Participatory Research^a^ ^a^Reproduced from Figure 1 in Cacari‐Stone and colleagues.^35^ ^(p1616)^

Perhaps partly in response to such expectations, Cacari‐Stone and colleagues (Table 1, model 4) have offered what might be referred to as a highly permeable logic model to link the co‐creation process (specifically, CBPR) to the policymaking cycle while also acknowledging the importance of the power‐knowledge axis (Figure 2).^35^ These authors propose that impact depends on aligning the contexts of the collaboration (political‐societal and specific collaborative histories) and partnership processes (eg, the extent to which decision making is equitable and the nature of leadership) with intermediate research and system or capacity outcomes, and more distally with health outcomes. They depict the policy process as iterative, nonlinear, and characterized by windows of opportunity. If we apply Weiss's terminology,^73^ co‐creation may influence this both instrumentally (by generating evidence) and interactively (through civic engagement).

**Figure 3:**
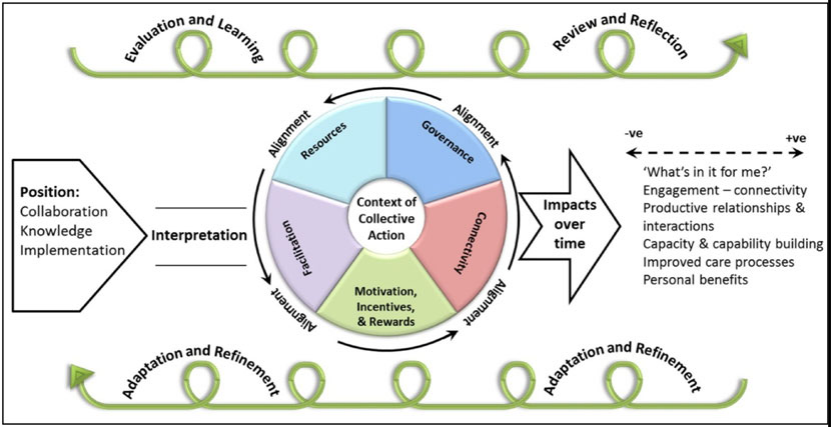
Realist Model of Impact in a Multi‐stakeholder Research Collaboration, Based on a National Evaluation of UK CLAHRCs^a^ +ve = positive, ‐ve = negative. ^a^Reproduced under terms of UK noncommercial government license from Rycroft‐Malone and colleagues.^66^

Other authors have chosen realism as a theoretical lens to help meet an expectation for “facts” in an inherently unpredictable system. Realist research seeks to produce more or less generalizable statements about what works for whom in what circumstances.^84^ Realist methods, based mostly on stakeholder interviews conducted over time, were used in a major national evaluation in the United Kingdom of the impacts of CLAHRCs, whose approach included elements of value co‐creation, experience‐based co‐design, and technology co‐design.^66^ Evaluators sought to tease out actors’ theories of change and explore how context shaped and constrained their efforts to achieve particular goals. The resulting impact model (Figure 3) encompasses all the key principles identified in previous work reviewed above: impact will be stronger and more enduring if the collaboration takes a systems perspective; frames research as a creative enterprise with human experience (particularly that of patients and staff) at its core; ensures an appropriate style of leadership; and emphasizes processes (especially relationships, interactions, sensemaking, and dialogue) as well as outcomes.

Jagosh and colleagues used a realist lens to study processes and outcomes of co‐creation in a systematic review of participatory research programs (Table 1, model 4).^62^, ^85^ This review confirmed the importance of relationship building, facilitation, and democratic program governance. More specifically, it centered on the notion of partnership synergy, defined as combining people's perspectives, resources, and skills to “*create something new and valuable together—a whole that is greater than the sum of its individual parts*.”^86^ ^(p318)^ Multi‐stakeholder partnerships are often characterized, at least initially, by conflict, but synergy may increase as co‐governing partners work together, leading to convergence of perspectives by progressive alignment of purpose, values, and goals and growth of mutual understanding and trust through what has been termed a “ripple effect.”^85^

In their review, Jagosh and colleagues suggest that it is this *process* of emerging partnership synergy, not the structures of governance per se, that is the key to success in CBPR.^36^ This construct may have wider applicability. Indeed, there may be parallels with what Janamian and colleagues have called the “interlocking” of academic and nonacademic stakeholders to generate impacts in the co‐created value chain.^65^ This process is described further in the case study that follows.

## Case Study: Co‐creation in a Primary Care “Beacon” Practice

We describe an example of the application of the value co‐creation model in a community‐based initiative in Queensland, Australia, which partnered research, service delivery, and professional communities. Following the “n of 1” case study approach of Stake,^87^ we deliberately present this case in narrative form. Further empirical details, including quantitative outcomes metrics and the perspectives of community stakeholders, are available in the additional articles referenced in this section.

In 2005, the University of Queensland and the local health board, Queensland Health, faced a problem—their mutually run general practice teaching facility was losing $800,000 annually and was destined for closure. Health care delivery to a vulnerable and underprivileged community was under threat. For the university this also meant the potential loss of a significant and long‐standing teaching facility; the health board faced a possible reputational and political risk. A small team of academics and executives from clinical backgrounds was tasked with finding a solution.

The initial team was attracted to the value co‐creation approach (model 1 in Table 1) because it appeared to allow a collaborative engagement with a focus on service transformation to create value for *all* end users.^30^, ^88^ The approach resonated with a shift in health care away from “quality improvement” (increasingly seen as aspirational and of questionable generalizable efficiency) and toward “value‐based health care,” in which the ideal of quality was explicitly aligned with the business case for achieving it and with quantifiable estimates of benefits, both human and financial.^89^, ^90^ The architects of the value co‐creation model emphasize that co‐creation is not about “build it and they will come” but rather “build it with them, and they are already there.”^37^ Faced with a stark economic picture and a network of health care providers who all sought to improve health outcomes but were not yet delivering these efficiently, the team found the idea of developing what Ramaswamy and Ozcan^37^ have called a “nexus of engagement platforms” oriented to delivering business success (“enterprise”) in a way that placed the patient (and staff) experience at the core of the endeavor and generated tangible value for all stakeholders (Figure 1) relevant and appealing.

The first step was to invite individual stakeholders (in this case, service users, primary and secondary care providers, funders, policymakers, and health bureaucrats) to be involved in co‐creating the value proposition and the network and processes through engagement and dialogue. A small design and planning group, comprising relevant organizational leaders known for their innovation and flexibility, was formed to review potential approaches and prepare a proposal for funders. They recommended a new practice model—a “beacon” practice—be established. The vision was for a community‐focused practice, premised on an ethos to support and extend the capacity of all primary care in the area and to better integrate service delivery locally between general practice, specialist services, and other state‐funded care. The beacon practice would be separated from individual funders and governed as its own not‐for‐profit company, with a board whose membership reflected the community it served and with a goal of restoring financial balance within a 3‐year time frame.

The 7‐person board comprised 2 nominees each from Queensland Health and the University of Queensland, a community member (the local member of parliament and recipient of complaints regarding health and social care delivery in the area), a representative with formal financial skills, and an independent chair with skills in strategic leadership and primary care reform. All board members were champions of care integration, community development, and service partnership. The board established a clinical advisory committee, with broader input from clinical care deliverers locally, and a research committee to inform and evaluate the new approach to care. Decision making was shared equally across the co‐creators, with consensus always the sought end result. Actions did not proceed until all co‐creator organizations were signed up.

As well as the need for good primary care access (already recognized as a priority), working groups identified other key health issues: long waits for outpatient assessment for patients with complex chronic disease and significant transport and health access difficulties for the community. Hospital clinicians were concerned about high (up to 40%) nonattendance rates for outpatient appointments in the area and expressed commitment to working with primary care clinicians on innovative solutions with shared resources. All groups engaged in robust debate as they sought to find agreement on the optimal path to community access, affordability, and service excellence.

Individuals invited to join these working groups were selected for having a personal commitment to a philosophy valuing innovation, multisector diversity, and patient‐centeredness. They became champions of the new approach within their own organizations and identified opportunities for improved care and funding efficiency to assist their individual executive and clinician communities in supporting the change. A significant feature of this collaboration was that stakeholders’ diverse organizational, fiscal, and governance challenges, opportunities, and requirements were discussed frankly and openly in all meetings and activities. All partners committed to an outcome where the service model was valued by all, even if not directly delivered by each.

Meetings and interactions were supported by real‐time data (such as aggregated patient clinical and access statistics, service costings, previous minutes and commitments, and staff and patient feedback). A subgroup was established to develop unique best practice clinical guidelines supporting chronic disease management across the care continuum (this required capacity building among primary care staff). More generally, the shared commitment to basing decisions on robust evidence inspired a series of systematic literature reviews addressing the evidence base for optimal practice organization,^91^ challenges to implementation of new service models in primary care,^92^ and governance models for primary‐secondary care integration.^93^ The program also required and supported a stream of empirical research, undertaken in parallel with the change effort, on the organizational challenges and patient and staff experience of the new model. Research activities included a development and pilot study of a primary care improvement tool,^94^ a qualitative evaluation of a clinical microsystems model in diabetes care,^95^ a quantitative study of the evidence‐practice gap in gestational diabetes follow‐up,^96^ and the development and evaluation of a community‐based diabetes surveillance and management program.^97^, ^98^, ^99^, ^100^

The many partner organizations brought different capabilities and connections and drew on these collaboratively to create the new practice, devolving some hospital functions to the community (eg, diabetes self‐management education and insulin stabilization) with the increased primary care capacity made possible via the beacon. Within 3 years, the beacon practice was revenue neutral and was partnering with local clinicians to care for complex care patients from the area with better outcomes and high satisfaction at significantly reduced cost. For example, diabetes control in patients served by the beacon practice improved over time; compared to neighboring sites, preventable diabetes‐related hospitalizations halved and metrics of patient satisfaction and empowerment increased.^98^, ^99^, ^100^

The partners continued to grow “ecosystems of capabilities” (see Figure 1) together. As the partnership has grown, trust has increased and relationships have strengthened (“partnership synergy”), allowing the collaboration to share robust data on both processes and outcomes and to make medium‐term strategic decisions (such as the continuation of applied research programs). The initiative created excitement within the broader health environment; it received various awards and attracted public interest, which generated further impetus through a “ripple effect.”^85^ A notable success was the receipt of both substantial Department of Health funding to extend the beacon model to other parts of the state and major research grant funding (from the Australian National Health and Medical Research Council) to develop a center of excellence in integrated care. Beacon practices are now being established in refugee health, chronic kidney disease, and maternity care.

The story of Queensland's primary care beacon practice included a number of established success features of the value co‐creation approach: creating a common vision oriented to delivering value for each partner member; flexible, outcome‐focused leadership; recognizing and respecting the very different cultures and perspectives of organizational stakeholders; committing to ongoing collaborative relationships rather than one‐off projects; and collectively celebrating wins and problem‐solving challenges. The research stream resonated with a key success principle gleaned from our literature review: a creative approach to research with a strong collaborative and ongoing partner involvement in design—and human experience at its core. Whilst the approach taken was explicitly aligned with model 1 in our taxonomy (see Table 1), it thus also had features of model 2 (experience‐based co‐design).

The program described in this case study was by no means smooth sailing. Both at the outset and periodically as it unfolded, stakeholders clashed at both clinical governance and executive meetings regarding the best way forward. Members were often very focused on their traditional ways of doing things, tending to default to historical roles, funding streams, and divisions of labor. Group leaders managed these conflicts proactively, using the guiding co‐creation engagement principles and a strong governance structure that aided power sharing and conflict management. A few initiatives took several meetings to reach consensus; though since the agreed priority was innovative care delivery that created value for all, partners were respectful of the need for this. They learned to define “value” more flexibly (the term might, for example, mean improved access or less tangible benefits, rather than direct care delivery).

Meeting chairs were selected for their leadership qualities, ability to identify and rise above “groupthink” (bland consensus was explicitly discouraged), and commitment to ensuring that potential challenges to new ideas were identified and vigorously discussed. They set an important ethos of constructive criticism and creative innovation, with the patient experience as the central focus.^31^ They recognized that if properly handled, conflict was not merely healthy and constructive, but an essential process in achieving successful change in a complex adaptive system.^85^ As partners worked together, trust was built through a growing understanding and valuing of one another's different positions and organizational requirements. Co‐creation champions looked for ways to neutralize territorial anxieties by identifying opportunities to deliver better local services together.

## Discussion

This article has described, via both a literature review and a supporting case study, the key principles required for effective co‐creation in community‐based research. These include (1) a systems perspective (assuming emergence, local adaptation, and nonlinearity); (2) the framing of research as a creative enterprise oriented to design and with human experience at its core; and (3) an emphasis on process, including the framing of the program, the quality of relationships, and governance and facilitation arrangements, especially power‐sharing measures and the harnessing of conflict as a positive and engaging force.

Being mindful of the above principles in designing and implementing co‐creation projects is likely to help maximize their success. Because co‐creation requires a systems perspective, a “logic model” mind‐set with inflexible goals will be less effective than an approach that acknowledges nonlinearity and encourages local adaptation as the program unfolds. Co‐creation models are not suited to all kinds of research. Rather, they appear to lend themselves to a particular kind of research—the systematic study of creative efforts oriented to improving human experience (a focus that has the potential to align stakeholders within a complex system). Both our own case example and others in the literature illustrate that “impact” efforts need to face internally as well as externally; they depend critically on the quality of relationships within the collaborative and the effectiveness of stakeholders’ ongoing negotiation of, and reflection on, the program's changing goals. Robust governance, skilled facilitation, relationship‐building efforts, and conflict management are all necessary (though as argued below, they may not be sufficient) to assure success.

Despite (and perhaps partly because of) the evident tensions and ongoing negotiations among the project's various stakeholders, the Queensland beacon practice appears to have been more successful than many other similar multi‐stakeholder co‐creation initiatives. In a recent empirical study of governance in a UK CLAHRC, for example, Fitzgerald and Harvey found that governance structures never “gelled” and that as the program unfolded, partners began to withdraw their commitment and funding.^101^ One reason for this was that once established, the network became divided into silos, each of which became very externally facing and focused on “knowledge translation” to audiences beyond the CLAHRC. There was marked duplication of effort and weak internal communication, to the extent that different teams within the CLAHRC had little idea what other local teams were doing. The authors concluded that externally facing knowledge translation measures are insufficient to ensure local uptake and impact of research findings; there also needs to be attention to the internal mobilization and negotiated utilization of knowledge *within* the network of participating stakeholders—a process they describe as a “balanced power” form of collaboration.^101^

Multi‐stakeholder research‐service collaborations are characterized by structural complexity and multiple competing interests and by pressure from various quarters to measure their activities and impacts and to demonstrate accountability. Power and conflict are prominent themes in published evaluations of these complex forms, which talk of “*colliding institutional logics,*”^102^ “*ambiguous loyalties … different interests… competing goals,*”^103^ and “*multiple accountabilities*.”^64^ Perhaps especially where commercial partners or government are involved, competing interests may loom large. Hinchcliff and colleagues distinguish between the sanitized written accounts of multi‐stakeholder interactions (“*draped in the formal collaborative language and procedures prescribed by funding agency protocols*”) and the reality in which “*participants … view each other pragmatically as consultants, clients or even competitors, rather than partners*.”^62^ ^(p126)^

Arguably, the structures of co‐creation are inherently unstable and may have unclear and/or shifting goals. Bennet and colleagues have used the metaphor of *collaborative entanglement* 104 (explored further by Phipps and colleagues^105^) to depict the conflict‐ridden, messy, unpredictable, and evolving interactions possible among stakeholders pursuing Mode 2 activity where structures and governance are suboptimal. As Van de Ven and Johnson's pragmatic theory predicts, the structures of co‐creation depend on skilled leadership, ongoing negotiation, and dedicated resources (time, expertise, money) to focus the salient features of reality and avoid descent into stalemate, ensuring that organizations deliver on agreed commitments.^62^, ^64^, ^67^

The relatively recent emergence of complex forms for co‐created research has, arguably, outstripped the pace of research into the optimum structures and types of governance that might support them. The value co‐creation literature views robust governance structures and co‐creative management systems as the key mechanisms for guiding the co‐creation process both within the enterprise and with external stakeholders.^37^ Such governance includes setting ground rules for co‐creative applications, defining early on who is responsible for what and to whom, and ensuring evenly distributed power constellations. This model depicts the co‐creation of value as dependent on high‐quality stakeholder interactions and the integration of resources among them—and this in turn depends on the formal and equitable distribution of power, for which active involvement of stakeholders is a requirement (since participants must carry the responsibility for the implementation and consequences of shared decisions).^106^

An alternative argument is that governance structures alone cannot assure the appropriate power‐sharing processes for the complex forms that now characterize community‐based research. Brown has argued from a Foucauldian perspective that the interaction between researchers and nonacademic stakeholders is complex and necessarily political; the structures of democratic interaction (representation, independent chairing, transparency, public debate, and so on) may or may not overcome the subtle and insidious use of power by vested interests (for example, in defining what kind of knowledge “counts” in key decisions).^82^ Indeed, Schmachtel has coined the term “rationalized myths” to depict the illusion of order and democracy that is inevitably created when a local collaboration is formalized; the shared narrative and supporting structures serve both to “*…[legitimize] the partnership's setup, yet [conceal] its complex, contradictory and antagonistic reality*.”^107^ ^(p1)^

These two contrasting framings—the one focusing on the formal distribution of power and the management of overt conflicts of interest, the other seeking to expose and question more covert influences—raise a key empirical question (but one that has no easy answer): to what extent was the success of the Queensland case study attributable to good governance processes (including the selection of leaders for particular personal qualities)? More generally, to what extent can such good governance processes alone assure the success of co‐creation partnerships in community settings?

To date, the literature on co‐creation, both within and beyond the health care field, has tended to be aligned with particular projects and programs. Less attention has been paid (at both a theoretical and an empirical level) to the co‐creation of policy and guidance—especially the processes for guiding and overseeing health research systems—to inform and shape how we define and judge research. In other words, the models set out in Table 1 and reviewed above are helpful in thinking through the co‐creation process *in* particular programs of research but have so far contributed little to the science *of* research—for example, the study of how research is (co‐)developed, funded, assessed, and evaluated. Given that the literature on “research *on* research” remains dominated by a “Mode 1” ethos and values, the applied science of co‐creation may yet take some time to establish a stronger and more coherent paradigm.

## Conclusion

This article has reviewed the literature on co‐creation in community‐based health care with a particular emphasis on societal impact and offered a worked example of one model in practice. Whilst we are not suggesting that our single example is statistically generalizable, both the literature review and the case study support the conclusion that it is not collaborative *structures* per se that add value in the research‐impact relationship. Rather, those who wish to reap the benefits of a co‐creation model should carefully note, and seek to apply, the key success principles advocated by this article, so as to avoid failure in the complex, important, and (sometimes) rapidly evolving partnerships that now exist between researchers and health service end users.

In conclusion, co‐creation models offer one approach to moving research out of the ivory towers and closer to the real world. As such, they have high potential for research impact, though such impact is by no means guaranteed. Among the key success principles for achieving societal impact from co‐creation models are embracing an adaptive “complex system” model of change and attending carefully to processes, relationships, and conflict management (though the extent to which these efforts will produce success is likely to depend on local contingencies). It surely follows that in order to capture the nonlinear chains of causation in the co‐creation pathway, impact metrics should evolve to reflect the dynamic nature and complex interdependencies of health research systems and address processes as well as outcomes.
